# Experimental data for effect of carbon black loading on tensile, hardness and rebound of magnetic iron filled natural rubber composites

**DOI:** 10.1016/j.dib.2019.104166

**Published:** 2019-06-21

**Authors:** R. Ismail, Nurul Husna Binti Rajhan, Hanizah Abdul Hamid, Azmi Ibrahim

**Affiliations:** a Institute of Infrastructure Engineering and Sustainable Management (IIESM), Universiti Teknologi MARA, 40450, Shah Alam, Selangor, Malaysia; b Faculty of Civil Engineering, Universiti Teknologi MARA, 40450, Shah Alam, Selangor, Malaysia

## Abstract

Generally, a base isolator is made up of alternate layers of steel and rubber. The idea of adopting magnetoreological elastomers (MREs) in base isolator systems was introduced in the past few years in order to improve the efficiency of base isolator systems. The article provides information on the mechanical corresponding to different carbon black loading loadings of 20 parts per hundred rubber (pphr), 40 pphr and 60 pphr in natural rubber compound. The mechanical dataset described the data from tensile, hardness and rebound test.

**Table:** Specifications table

Subject area	*Civil and Structural Engineering, Composite, Material Science Engineering*
More specific subject area	*Mechanical Properties, Polymer Physics,*
Type of data	*Table, text file, graph, figure*
How data was acquired	*Tensile Instron Machine (Tempro-5569), International Rubber Hardness Tester (IRHD-H14), Wallace Dunlop Tripsometer*
Data format	*filtered, analyzed*
Experimental factors	*The cure characteristic parameters measured include scorch time, cure time, maximum torque and torque differences. The compound batches had been left at least 16 hours before being cut and tested* *The curing temperature was conducted at 150 °C. The test pieces had been conditioned at 23 °C for at least 3 h before conducting the testing.*
Experimental features	*The samples was compounded by incorporation of different carbon black loadings of 20 parts* per *hundred rubber (pphr), 40 pphr and 60 pphr. Five dumbbell test pieces of MRE samples were prepared to determine the tensile strength and the average result from the five test pieces was used. Two round shape test pieces were prepared for hardness and rebound tests.*
Data source location	*Data obtained from the material laboratory, Faculty of CIVIL Engineering and Faculty of Applied Science, Universiti Teknologi MARA.*
Data accessibility	*All the data are in this article as presented.*
Related research article	*Rajhan, N. H., Hamid, H. A., Ibrahim, A., & Ismail, R. (2016). Experimental study on mechanical properties of magnetorheological elastomer. Jurnal Teknologi, 78(5–4)**[1]**.*

**Table:** 

**Value of the data** • The data presented shows that the three different carbon black loadings of 20 parts per hundred rubber (pphr), 40 pphr and 60 pphr used increased the mechanical properties of the natural rubber compound. • In order to create the magnetic property in this rubber compound, carbonyl iron powder was added into the MREs. • These data have important signiﬁcance for the basic parameters for the design of elastomeric bearings used for isolation of structure from external vibration like earthquake. • Data presented here could be helpful in further research on magnetic rubber modification of carbon black. Due to rubber properties are depending on compounding ingredients especially vulcanization system, type and amount of filler and other special ingredients for better performance.

## 1 Data

Data presented in this article was used to investigate the performance of MRE composites due to effect of carbon black loading. The mechanical test through tensile test, hardness test and rebound test. The data are focused on the mechanical properties of magnetic iron filled natural rubber composites.

The data of the tensile properties are tabulated in Table 1 and plotted in Fig. 7, Fig. 8, Fig. 9. The Effect of carbon black loading on rebound resilience tabulated in Table 2 and plotted in Fig. 11. The data for hardness test are tabulated in Table 3 (The thickness of hardness test pieces) and Table 4 (Results of hardness).

**Table 1:** Tensile test results.

Sample	Tensile strength (MPa)	Elongation at break E_b_ (%)	Modulus 100 (MPa)	Modulus 300 (MPa)
CB00	22.97	801.32	0.99	2.01
CB20	14.52	737.46	1.16	2.94
CB40	23.10	357.48	1.50	4.36
CB60	19.03	816.20	1.61	5.42

**Fig. 1:**
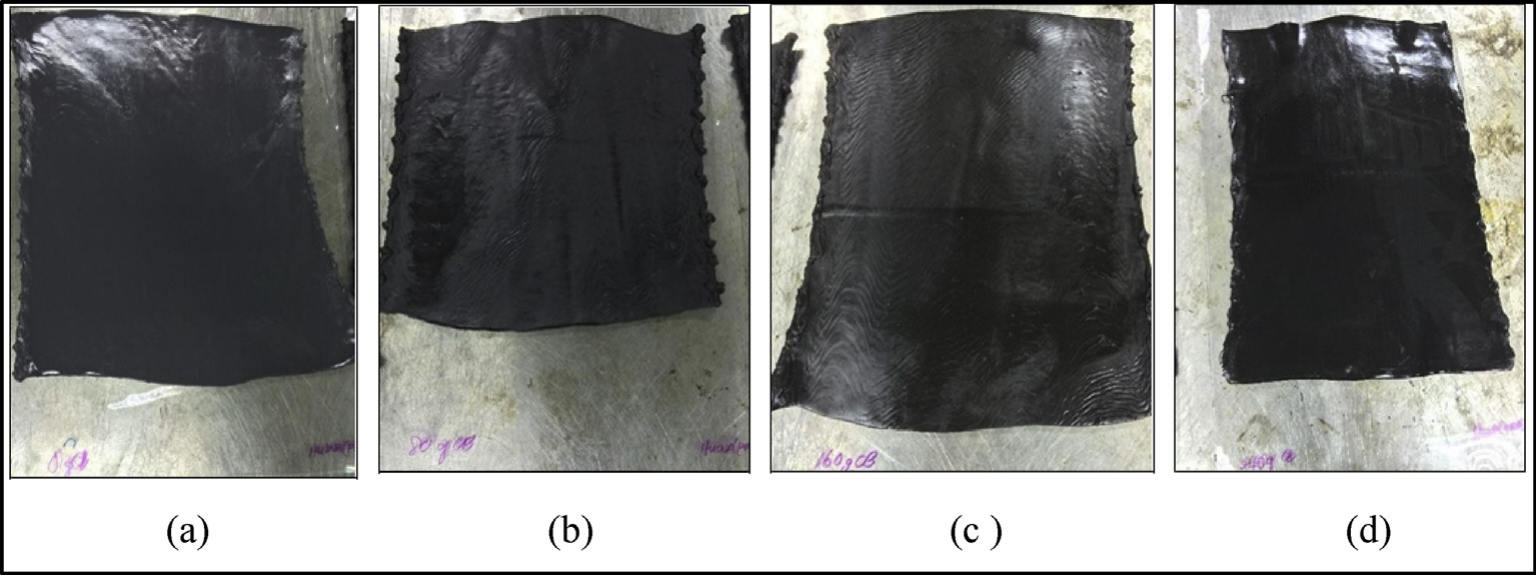
Green stock of MRE compound with different loadings of carbon black (a) 0, (b) 20, (c) 40, and (d) 60 pphr.

**Fig. 2:**
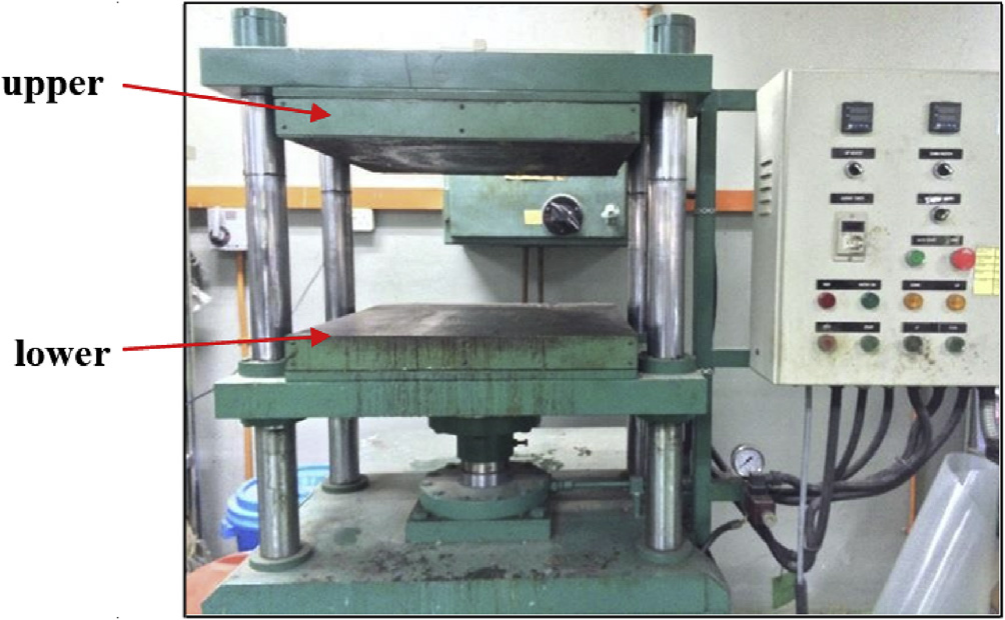
Hot press machine.

**Fig. 3:**
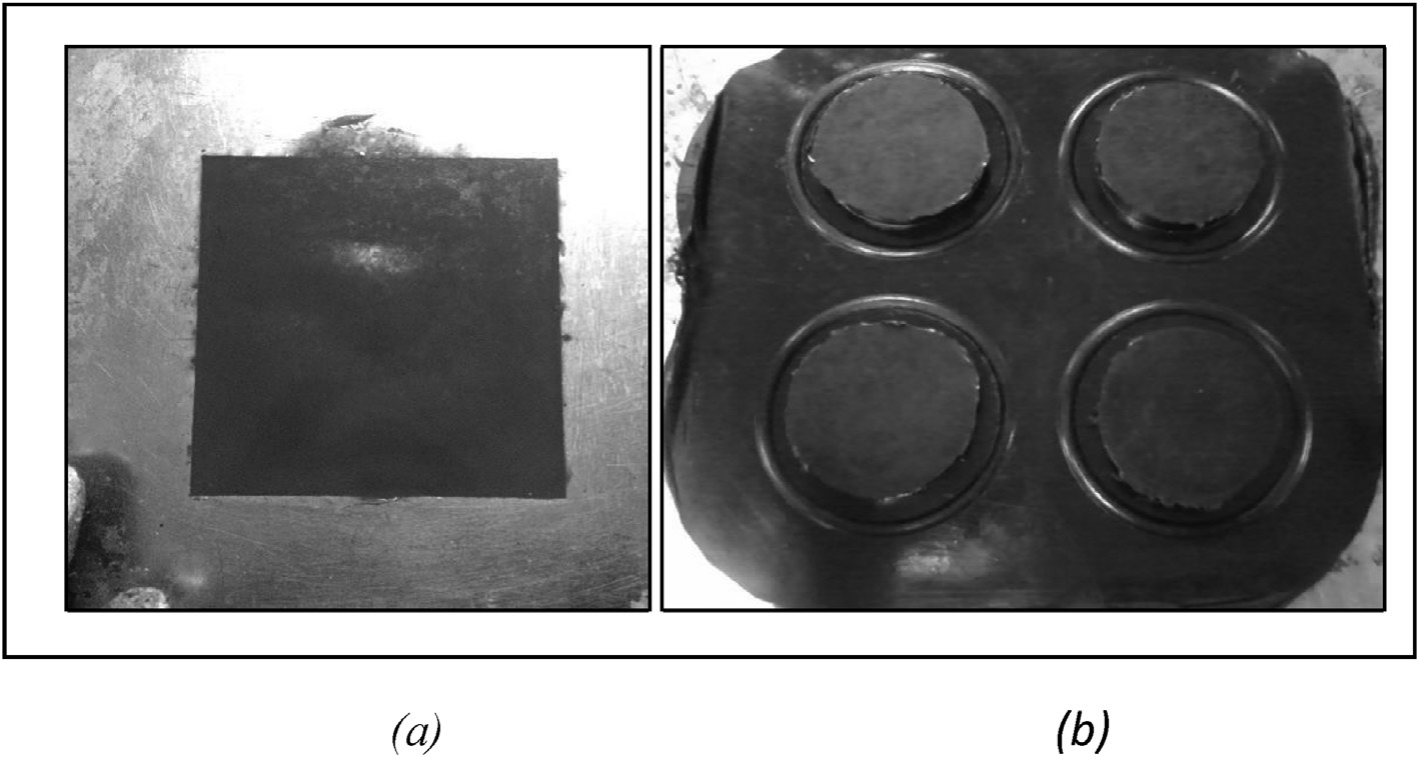
Sample after vulcanization using (a) square mould (b) round mould.

**Fig. 4:**
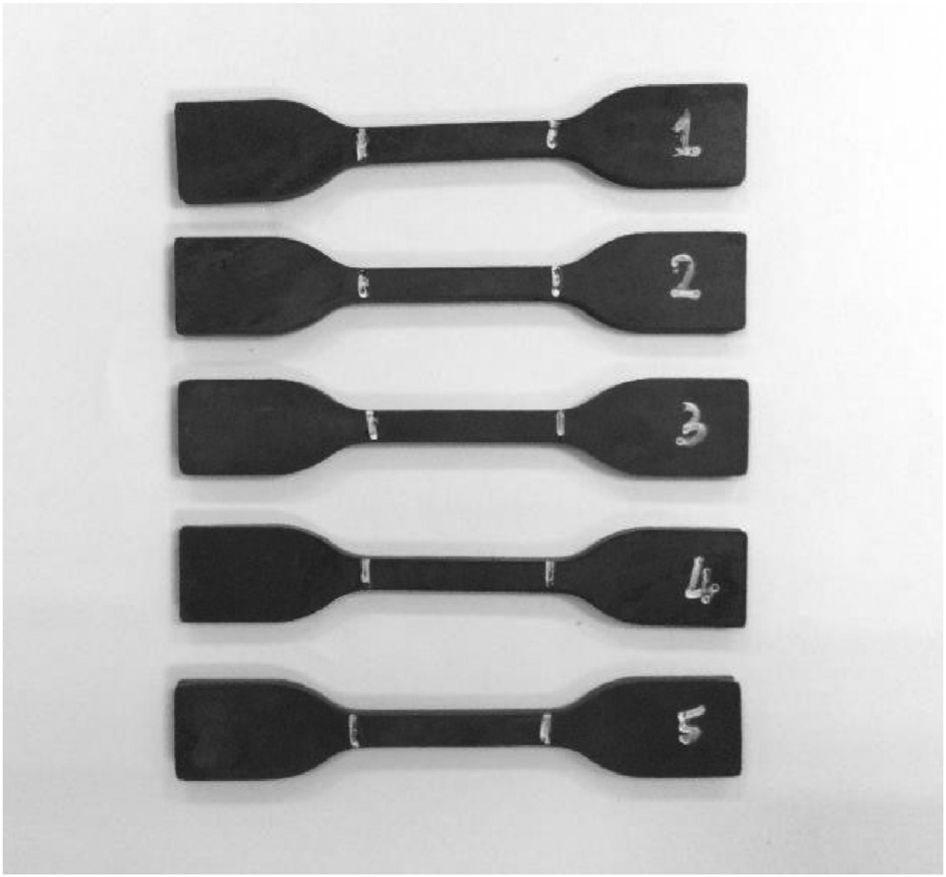
Dumbbell test pieces.

**Fig. 5:**
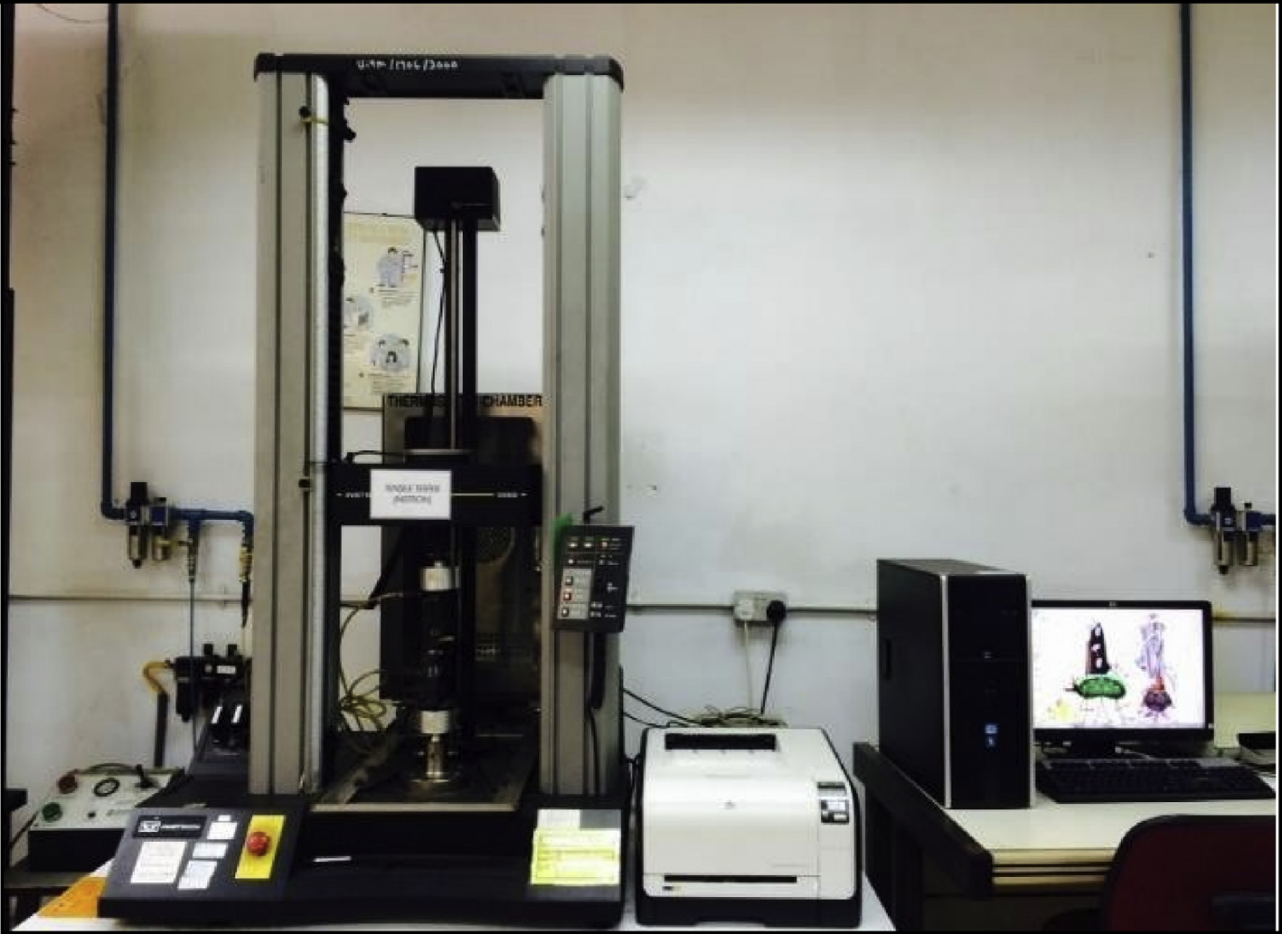
Set up the tensile instron machine.

**Fig. 6:**
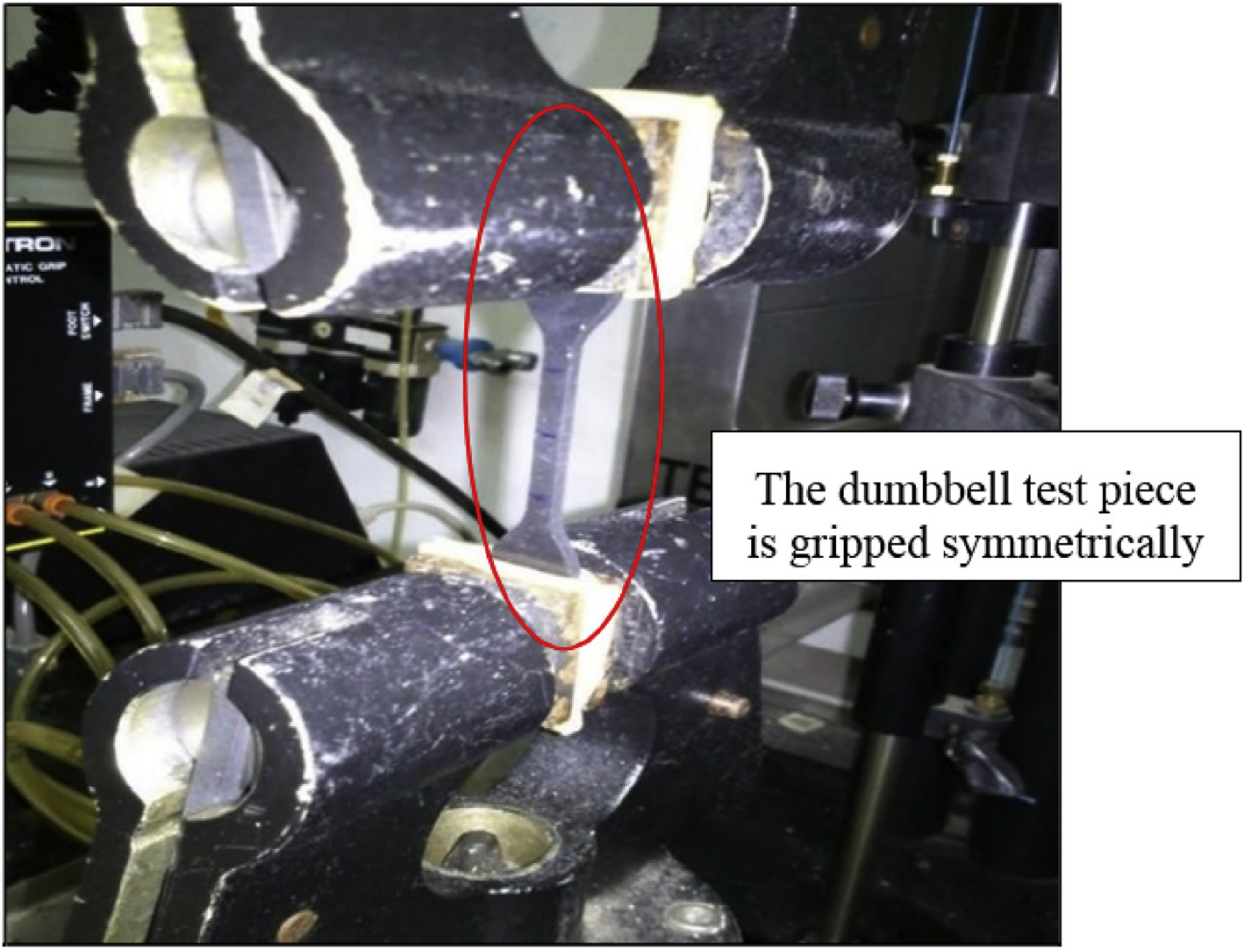
Insertion of test pieces.

**Fig. 7:**
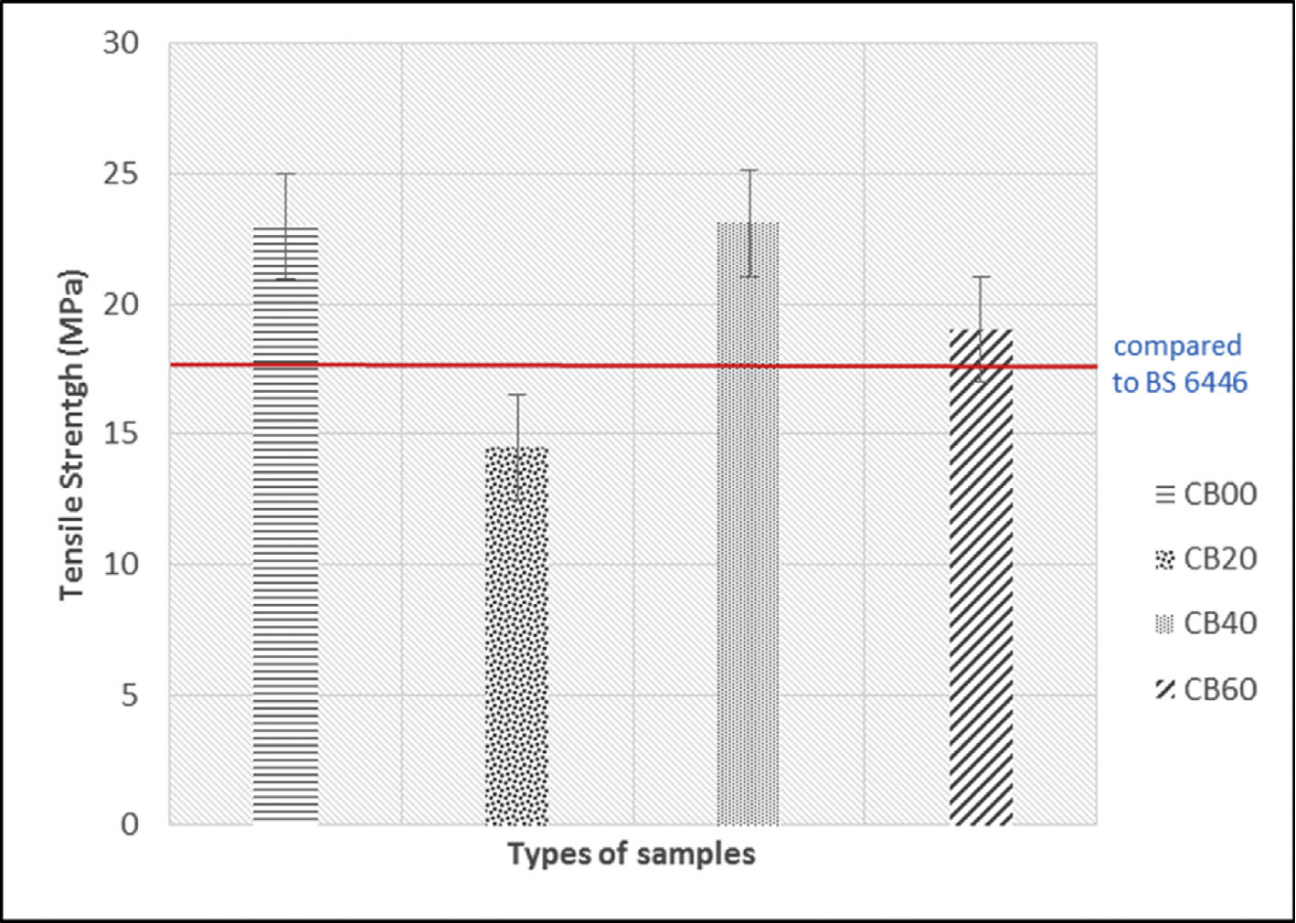
Effect of carbon black loading on tensile strength of MRE.

**Fig. 8:**
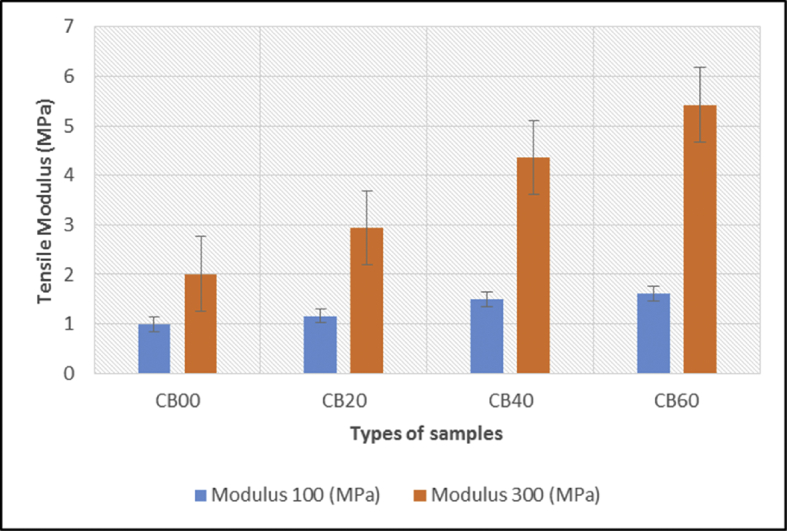
Carbon black loading at 100% (M100) and 300% (M300) elongation.

**Fig. 9:**
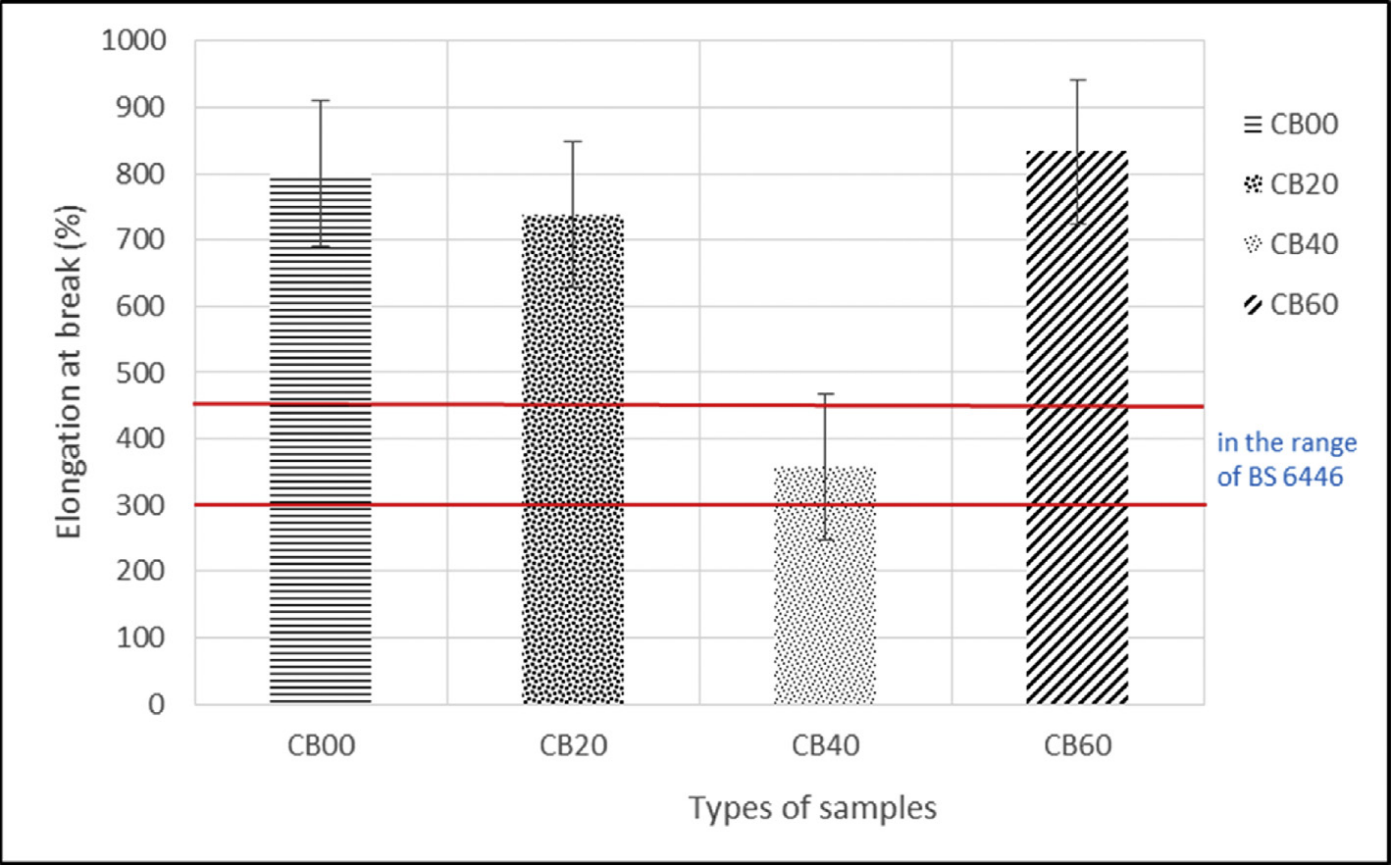
Effect of carbon black loading on elongation at break.

**Table 2:** Effect of carbon black loading on rebound resilience.

Sample	Sample Number	Results (%)				
		Reading 1	Reading 2	Reading 3	Median	Average
CB00	CB00-1	73.41	74.91	74.53	74.53	74.62
	CB00-2	74.71	75.59	74.71	74.71	
CB20	CB20-1	75.09	75.56	75.47	75.47	75.43
	CB20-2	75.56	74.53	75.39	75.39	
CB40	CB40-1	71.30	72.44	75.59	72.44	73.20
	CB40-2	72.03	74.34	73.96	73.96	
CB60	CB60-1	69.07	70.53	70.17	70.17	70.55
	CB60-2	69.52	70.93	71.57	70.93

**Table 3:** The thickness of hardness test pieces.

Sample	Sample No.	Thickness (mm)			
		Reading 1	Reading 2	Reading 3	Average
CB00	CB00-1	6.97	6.98	6.99	6.98
	CB00-2	7.00	6.97	6.95	6.97
CB20	CB20-1	7.11	7.07	7.03	7.07
	CB20-2	7.00	7.00	7.00	7.00
CB40	CB40-1	7.02	7.06	7.10	7.06
	CB40-2	7.11	7.08	7.04	7.08
CB60	CB60-1	7.02	7.03	7.05	7.03
	CB60-2	7.07	7.04	7.00	7.04

**Table 4:** Results of hardness.

Sample	Sample No.	Results (IRHD)				
		Reading 1	Reading 2	Reading 3	Median	Average
CB00	CB00-1	42.5	43.0	42.5	42.5	42.3
	CB00-2	41.0	43.0	42.0	42.0	
CB20	CB20-1	50.0	49.5	49.0	49.5	49.5
	CB20-2	49.0	49.5	50.0	49.5	
CB40	CB40-1	55.5	55.5	55.5	55.5	55.5
	CB40-2	56.0	54.5	55.5	55.5	
CB60	CB60-1	69.0	66.5	65.0	66.5	68.0
	CB60-2	70.0	69.0	69.0	69.5	

For further interpretation and discussion of the dataset, the reader is referred to the research data article [1].

## 2 Design, materials, and methods

### 2.1 Materials

Elastic matrix and magnetic particles are the main ingredients of MRE. In this experiment study, Standard Malaysian Rubber (SMR) L grade natural rubber was chosen as matrix based MRE. In order to develop the MRE compounds, carbonyl iron particles with The diameter and density of the iron particle are in range of 6–9 μm and 7.86 g/cm^3^, respectively were purchased from Sigma-Aldrich Sdn. Bhd. (M). Carbon black N220 was used as the reinforcing filler of the MRE compound which has been varied with addition of 20pphr, 40pphr and 60.

Other materials such as zinc oxide (ZnO), stearic acid and sulphur are also required as the basic ingredients of compounding unfilled rubber or filled rubber. In rubber standard compounds, (ZnO) and stearic acid have been used as activator and co-activator respectively. Cyclohexyl benzothiazolesulfenamide (CBS) and tetramethylthiuram disulphide (TMTD) are the accelerator and additives that had been selected in order to increase the properties of elastomers. Besides that, they were added as to help the vulcanization system.

### 2.2 Experimental design and methods

The batch of MRE compounds are named as CB00, CB20, CB40 and CB60. All quantities are expressed in parts per hundred parts of rubber (pphr). The compounding process of a batch mass of MRE was made by following BS ISO 2393 [2]. Fig. 1 illustrates the MRE compounds were obtained in sheets and conditioned at 23±°C for 24 hours before cure assessment.

The compounding process of MRE development was done using two roll mills and a conventional vulcanization system. The cure assessment of MRE composites was determined by Rheometer 100 The MRE final samples were vulcanized in square and round shape mouldings. For square mould, 60 g was required, whereas 16 g for round mould as can be seen in Fig. 2 The temperature was set at 150 °C for each sample. The final samples of MRE after compression moulded are shown as in Fig. 3.

In order for the material to be used for civil engineering applications, the MRE compound should satisfy and achieve the following general performances and quality control requirements according to BS ISO 6446 [3].

#### 2.2.1 Tensile test

Tensile properties of specimens were measured according ASTM D 412 [4], The dumbbell test pieces were tested by using an Instron Universal Tensile Machine equipped with 500N load cell at a static crosshead speed of 500 mm/min according to BS ISO 37 [5]. Fig. 4 shows the shape of dumbbell test pieces following the standard BS ISO 37 [5]. The Instron Tensile Machine as in Fig. 5 was set up. After that, the dumbbell test piece was manually attached at the clipper of tensile machine. Referring to Fig. 6, the ends of the dumbbell test pieces was ensured to be gripped symmetrically. Thus, the tension was uniformly distributed over the cross-section.

#### 2.2.2 Rebound test

**Fig. 10:**
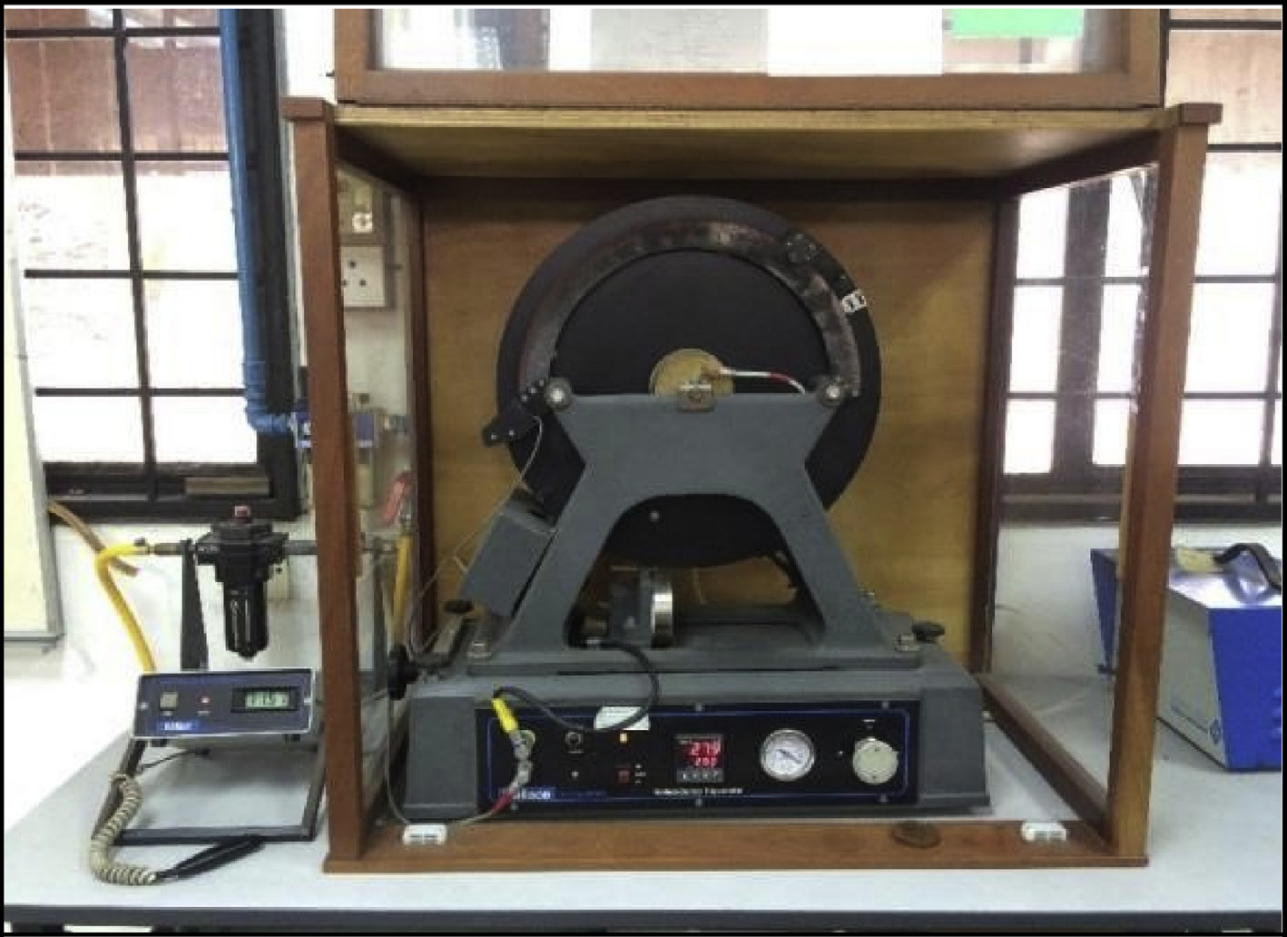
Dunlop Tripsometer at the laboratory.

Fig. 10 shows a Dunlop Tripsometer, which is the apparatus that used for determination of rebound resilience in this present study. The 4 mm thick test pieces and test piece holder were ensured clean. Method B of BS 903-A8 [8] is the reference to this test.

**Fig. 11:**
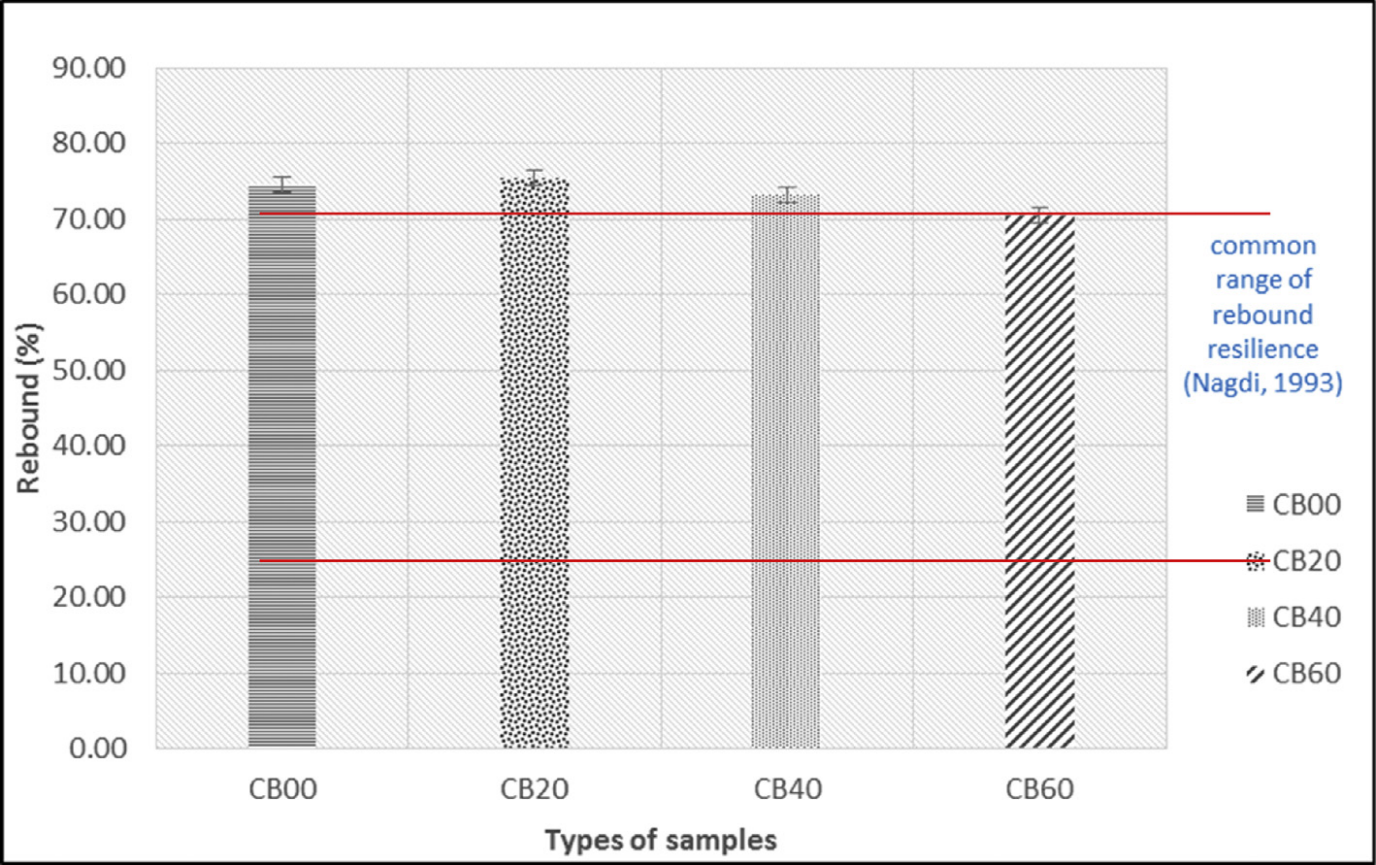
Histogram on rebound resilience (%) at different carbon black loading.

#### 2.2.3 Hardness test

**Fig. 12:**
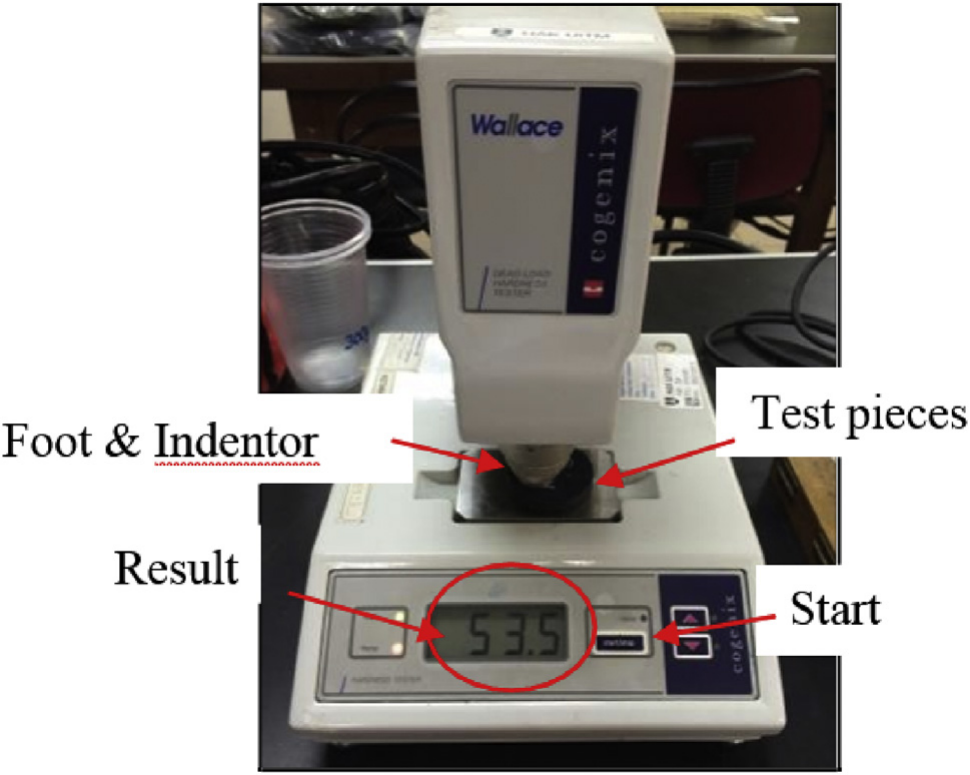
An automated dead load hardness tester.

According to BS ISO 48 [6], hardness is measured from the depth of indention of a spherical indentor, under a specified force, into a rubber piece. This type of testing is very simple and easy to be conducted. In addition, it is a non-destructive test. An International Rubber Hardness Tester (IRHD) (Refer Fig. 12), was used to measure hardness test for MRE samples. The standard test pieces were prepared according to BS ISO 23529 [7]. The thickness is about adequately 8 mm–10 mm thick and the thinnest should not be less than 2 mm thick. All surfaces of the test pieces should be flat and parallel.
